# Mutation hotspots in cis-regulatory regions in cancer

**DOI:** 10.18632/oncoscience.325

**Published:** 2016-11-23

**Authors:** Rebecca C. Poulos, Jason W. H. Wong

**Affiliations:** Prince of Wales Clinical School and Lowy Cancer Research Centre, UNSW Australia, Sydney NSW 2052, Australia

**Keywords:** somatic mutations, gene regulatory mutations, cancer, DNA repair

## INTRIGUING RESULTS

In recent years, somatic mutations in *cis*-regulatory elements of cancer genomes have become a focus of much research. A landmark discovery occurred in 2013, in which recurrent somatic mutations were identified in the promoter of the key cancer-associated gene, *TERT* (reviewed in [1]). In the search for other highly recurrent *cis*-regulatory mutations which may serve as novel driver events, two papers, published in 2014 [2, 3], revealed somewhat surprising results. These studies investigated large cohorts of cancer genomes and found that, despite identifying many recurrent promoter mutations, few could be associated with gene expression changes. Of those that did alter gene expression, many of their target genes did not have strong links to cancer development. We also published similar unexpected findings in a genome-wide survey of promoter mutations in the melanoma cell-line, COLO829 [4]. The study showed that while some regulatory mutations can alter promoter activity (∼17% of mutant promoter regions surveyed), one such mutation that was recurrent (∼4.4%) in other melanomas was not associated with altered gene expression in actual cancer samples [4]. Remarkably, we additionally observed that of the 14 remaining promoter mutations surveyed to not alter promoter activity, five mutations were also recurrent in melanoma samples. Together these articles raised the question of why there are such high rates of recurrence among promoter mutations if many do not appear to arise due to their oncogenic ability to alter gene expression.

## A MECHANISTIC EXPLANATION

Earlier this year, two publications in Nature provided a potential explanation for these otherwise intriguing results. In Perera, et al. [5], we showed, together with Sabarinathan, et al. [6], that nucleotide excision repair (NER) enzymes appear to be prevented from accessing DNA and repairing mutagenic DNA lesions by the presence of transcription factors bound to DNA. Specifically, XPC, a protein involved in NER, is unable to recognise DNA damage and recruit the enzymes involved in this repair process. We observed high rates of promoter mutations in a number of cancers which utilise NER, with particularly high numbers in melanoma, lung and ovarian cancers. We further showed in Perera et al. [5], that NER is specifically inhibited at sites of binding of the transcription initiation complex, accounting for the high rates of mutations at promoter, as opposed to enhancer, elements. Typically, recurrent mutations are deemed more likely to be cancer driver events. However, these findings provide an alternate mechanistic explanation which can account for the highly localised accumulation of mutations in the promoters of many cancer genomes. Thus, recurrent mutations in these elements may not necessarily be cancer drivers, nor ought they to always be expected to change gene expression.

## NEXT STEPS IN THE SEARCH

Given the high prevalence across cancer cohorts of the *TERT* promoter mutations, it would be surprising if there are not many other examples of *cis*-regulatory driver mutations in cancer genomes. However, as some genome-wide searches have already been conducted using large cohorts, it is unlikely that events of such astoundingly high recurrence across different cancer types, as the *TERT* promoter mutations, will yet be identified. We consider it more likely instead, that oncogenic *cis*-regulatory mutations will next be found either at low prevalence or perhaps entirely unique to individual cancer samples. If this is the case, researchers must be able to accurately model the likelihood of occurrence of specific somatic mutations in order to identify true driver from passenger mutations. To date, numerous factors have been shown to affect mutation load including replication timing, chromatin organisation and nucleotide composition (reviewed in [7]). Our findings [5], together with those of Sabarinathan, et al. [6], further improve our understanding of how mutations accumulate at different regions of the cancer genome. The development of statistical models that take into account factors that affect DNA lesion formation and DNA repair will ultimately provide a better prediction of whether specific mutations have arisen as a result of clonal selection within a cancer sample.

Nevertheless, even with improved models to predict mutation occurrence, to prove that a mutation is functional, *in vivo* characterisation will be necessary. Currently, the correlation of mutations with gene expression, even at the patient-specific level, is hampered by complications of inaccurate measurements of gene expression within a heterogeneous population of cancer cells which are often highly contaminated by stromal cells. Single cell analyses are a potential solution to overcome the tumour heterogeneity problem. This can be done through the application of recently developed methods for the sequencing of the genome and transcriptome, such as DR-seq [8], in single cancer cells. By utilising such methods, researchers may begin to develop a clearer picture of the contribution of *cis*-regulatory mutations to changes in gene expression and ultimately, to cancer development.
